# Stage-specific predation and functional response of predatory mites to greenhouse thrips (*Heliothrips haemorrhoidalis*) on avocado

**DOI:** 10.1007/s10493-026-01144-9

**Published:** 2026-05-07

**Authors:** Junlin Cao, Zhi-Qiang Zhang

**Affiliations:** 1 School of Biological Sciences, University of Auckland, 3A Symonds Street, Auckland, 1010 New Zealand; 2 Bioeconomy Science Institute Maiangi Taiao, 231 Morrin Road, Auckland, 1072 New Zealand

**Keywords:** Predatory mites, Stage-specific predation, Pprey preference, Biological control

## Abstract

The greenhouse thrips *Heliothrips haemorrhoidalis* is a major pest of avocado (*Persea americana*), causing significant leaf damage and yield losses worldwide. Biological control using predatory mites offers a potentially sustainable alternative to chemical management, yet the effectiveness of different mite species and the role of prey developmental stage remain poorly understood. In this study we compared the predation capacity of four phytoseiid mites—*Amblydromalus limonicus*, *Amblyseius herbicolus*, *Amblyseius lentiginosus*, and *Neoseiulus cucumeris*—on first- and second-instar larvae of *H. haemorrhoidalis* under controlled laboratory conditions. No-choice experiments revealed significant differences (*p* < 0.001) among predator species and thrips stages, with *A. limonicus* exhibiting the highest predation rates and a strong bias towards first-instar thrips, while the other mite species showed negligible thrips consumption. Choice experiments further demonstrated that *A. limonicus* exclusively preyed on first-instar thrips when both larvae instars were simultaneously available, confirmed by Manly’s preference index (Manly’s α = 1). Functional response analysis indicated a Type II functional response of *A. limonicus* feeding on first-instar thrips, characterised by a high attack rate at low prey densities and saturation at higher densities due to handling time constraints. These preliminary 24 h laboratory results indicate pronounced stage-specific predation under the conditions tested and show that *A. limonicus* performed better than the other predatory mite species examined. However, because the experiments included no predator-free controls, the findings should be interpreted cautiously and future studies under field or orchard conditions are needed to demonstrate the effectiveness of *A. limonicus* in *H. haemorrhoidalis* biocontrol.

## Introduction

Avocado (*Persea americana* Mill.) is an economically important subtropical crop that has gained increasing global popularity due to its high nutritional value and wide range of culinary and industrial uses (Ayala Silva and Ledesma 2014; Majid et al. 2020; Nelson Ceballos-Aguirre et al. 2021; Talavera et al. 2023). However, avocado production is frequently constrained by arthropod pests that damage leaves and fruits, leading to reduced yield and market quality (Erichsen and Schoeman 1992; Hoddle et al. 2003).

An important thrips species associated with avocado is the greenhouse thrips, *Heliothrips haemorrhoidalis* (Bouché) (Thysanoptera: Thripidae). It is widely distributed across avocado-growing regions worldwide, including New Zealand (Larral et al. 2018; Logan et al. 2021; Stevens et al. 1999). Thrips are particularly challenging pests due to their small body size, rapid population growth, and cryptic feeding behaviour, which complicate effective monitoring and management (Hoddle et al. 2003). Feeding by *H. haemorrhoidalis* causes direct damage to avocado leaves through cell rupture and sap extraction, leading to leaf discoloration, reduced photosynthetic capacity, premature leaf drop, and (under high infestation levels) severe defoliation and yield losses (Wysoki et al. 2002). Management of greenhouse thrips in avocado orchards has traditionally relied on broad-spectrum insecticides (Stevens et al. 1999). However, repeated chemical applications can disrupt communities of natural enemies, promote pest resurgence, and increase the risk of insecticide resistance (Hill et al. 2017; Roubos et al. 2014).

These control limitations have driven growing interest in environmentally sustainable alternatives such as biological control. Among the natural enemies associated with greenhouse thrips, the larval endoparasitoid *Thripobius semiluteus* has received particular attention as a biological control agent of *Heliothrips haemorrhoidalis*, and establishment after release has been documented in California and New Zealand orchard systems (Jamieson et al. 2008). Predatory mites in the family Phytoseiidae (Acari: Mesostigmata) are among the most widely used biological control agents in horticultural systems and may help bridge this gap; however, previous studies have reported variable and often limited efficacy of different phytoseiid species against thrips (Tixier 2018; McMurtry et al. 2013). The success of predatory mites in suppressing thrips populations can vary substantially among species and is strongly influenced by the developmental stage of the prey, highlighting the need for stage-specific evaluation of predator-prey interactions (Vangansbeke 2015).

This study explored the biocontrol potential of four predatory mite species—*Amblydromalus limonicus* (Garman & McGregor), *Amblyseius herbicolus* (Chant), *Amblyseius lentiginosus* Denmark & Schicha, and *Neoseiulus cucumeris* (Oudemans)—against greenhouse thrips in a series of three laboratory experiments. These species were selected because some of them have shown predation or control potential against other thrips species in previous studies. On greenhouse cucumber infested with western flower thrips, *Frankliniella occidentalis*, comparative trials showed strong interspecific differences among phytoseiid predators, with *A. limonicus* among the most effective species tested, whereas *N. cucumeris* was less effective (Messelink et al. 2006). Laboratory studies on *Sericothrips staphylinus* likewise showed that *A. limonicus* had the highest predation and oviposition rates, *Am. herbicolus* showed intermediate performance, and *N. cucumeris* the lowest; a subsequent functional-response study further confirmed that *Am. herbicolus* can prey on this thrips species (Lam et al. 2019, 2021). Together, these studies indicate that phytoseiid predators can differ markedly in their performance against thrips and support a comparative evaluation of their capacity to attack *H. haemorrhoidalis*. *Amblyseius lentiginosus* was included as an additional phytoseiid for broader comparison under the same experimental conditions. However, the effects of these four predators on *H. haemorrhoidalis* have not previously been investigated.

## Approach

First, we conducted a no-choice predation experiment to assess the ability of different predatory mite species to attack and consume specific developmental stages of *H. haemorrhoidalis* under controlled laboratory conditions (Nielsen et al. 2014). By offering predators only a single prey stage, these assays allowed direct comparison of predation capacity among predator species without the influence of prey selection (Yang et al. 2025).

Second, we conducted a choice experiment because we reasoned that predation patterns observed under the no-choice condition might not reflect predator behaviour when multiple prey stages are simultaneously available. Because early thrips instars differ from later instars in size, mobility, and vulnerability, predators may actively select among prey stages under choice conditions (Beretta et al. 2024; Yang et al. 2025).

Third, we conducted a functional response analysis that aimed to characterise the response of predator mites to the density of first-instar larvae of *H. haemorrhoidalis* under controlled laboratory conditions. By quantifying prey consumption across a range of initial prey densities, this experiment sought to determine functional response type and estimate key predation parameters, providing mechanistic insight into density-dependent feeding dynamics relevant to biological control applications (Holling 1959; Juliano 2020).

## Materials and methods

### Study organisms and rearing conditions

Predatory mites used in this study included *Amblydromalus limonicus* (hereafter *A. limonicus*), *Amblyseius herbicolus* (hereafter *Am. herbicolus*), *Amblyseius lentiginosus* (hereafter *Am. lentiginosus*,) and *Neoseiulus cucumeris* (hereafter *N. cucumeris*). All predatory mite species were originally collected from avocado and plum leaves in Rototuna, Hamilton, New Zealand, in March 2024.

Predatory mites were maintained under controlled laboratory conditions at 25 ± 1 °C, 80 ± 5% relative humidity (RH), and a 16:8 h (hour) light: dark photoperiod at the Bioeconomy Science Institute Maiangi Taio (BSI), St Johns, Auckland, New Zealand (Zhang and Zhang 2021). All species were reared using identical experimental set-ups. Each culture consisted of a Petri dish placed on a plastic sheet, which was positioned on a slightly larger water-saturated sponge to provide continuous access to moisture while preventing mite escape (Wang et al. 2024). This set-up ensured adequate hydration and minimised contamination between cultures.

The dry fruit mite *Carpoglyphus lactis* was used as prey for maintaining predatory mite colonies and was obtained from a commercial supplier (Bioforce Limited, Karaka, Auckland, New Zealand). *Carpoglyphus lactis* was reared in 250 mL plastic containers with screw caps fitted with mesh-covered ventilation holes. The rearing medium consisted of a mixture of wheat bran, dry yeast, and icing sugar. The cultures were maintained at 25 ± 1 °C, 80 ± 5% RH, and a 16:8 h light: dark photoperiod, following standard protocols (Wang et al. 2024).

Wheat bran medium containing *C. lactis* was added regularly to predatory mite cultures as a food source. Four folded square plastic sheets (approximately originally 10 mm on each side) were placed in each culture to provide refuge and oviposition sites for predatory mites.

To establish age-synchronised cohorts for experiments, eggs (< 24 h old) of each predatory mite species were collected and transferred to new cultures supplied ad libitum with C. lactis. Eggs were obtained by placing short sewing threads (approximately 15 mm in length) into stock cultures overnight, which served as oviposition substrates. All adult predatory mites used in experiments were young adult females < 7 d after emergence (Zhang and Zhang 2021).

## Experimental arenas

All experiments used modified Munger cells as described by Wang et al. (2024). For the pre-test starvation treatment, individual predators were held in small plexiglass cells consisting of two transparent plexiglass slides (38 mm × 25 mm × 2 mm). The cell contained a conical aperture (6 mm in diameter at the top and 10 mm at the bottom). A slice of old avocado leaf was placed between the two plexiglass slides as a substrate for the thrips and mites, and four layers of filter paper were positioned beneath the avocado leaves to provide a water reservoir. The entire cell assembly was secured using a pair of metal clips. The top of the conical aperture was sealed with a layer of food wrap, into which five evenly spaced ventilation holes were pierced using a size 0 insect pin.

## No-choice test

For each predatory mite species, two prey-stage treatments were established using greenhouse thrips: first-instar (I) and second-instar (II) larvae. Predator species (PS) was treated as a four-level factor (PS1–PS4), representing *A. limonicus*, *Am. herbicolus*, *Am. lentiginosus*, and *N. cucumeris*, respectively, and prey stage was treated as a two-level factor (I vs. II). Each predator-prey-stage combination was replicated 10 times (i.e., 10 replicates for PS1–I, PS1–II, …, PS4–I, and PS4–II), giving a total of 8 treatment combinations and 80 experimental replicates overall.

Before each trial, a single adult female predatory mite (young adult; < 7 d after emergence; mated) was transferred into an empty experimental arena and starved for 24 h to standardise hunger levels. During the starvation period, water was supplied via four layers of moist filter paper to prevent desiccation (Summerfield et al. 2024). Following starvation, five thrips individuals of a single developmental stage (either I or II) were introduced into the arena. The starved predator was then placed in the centre of the arena, which was immediately sealed with food wrap to prevent escape while allowing ventilation. Water was again added to the filter paper to maintain humidity and leaf freshness throughout the experimental period. At the end of the 24 h exposure period, the number of thrips consumed was recorded. Prior to the assays, a preliminary run of predator-free control (8 to 10 replicates) under the same experimental conditions showed zero mortality of thrips larvae on leaves in the cell.

## Choice test

**Fig. 1:**
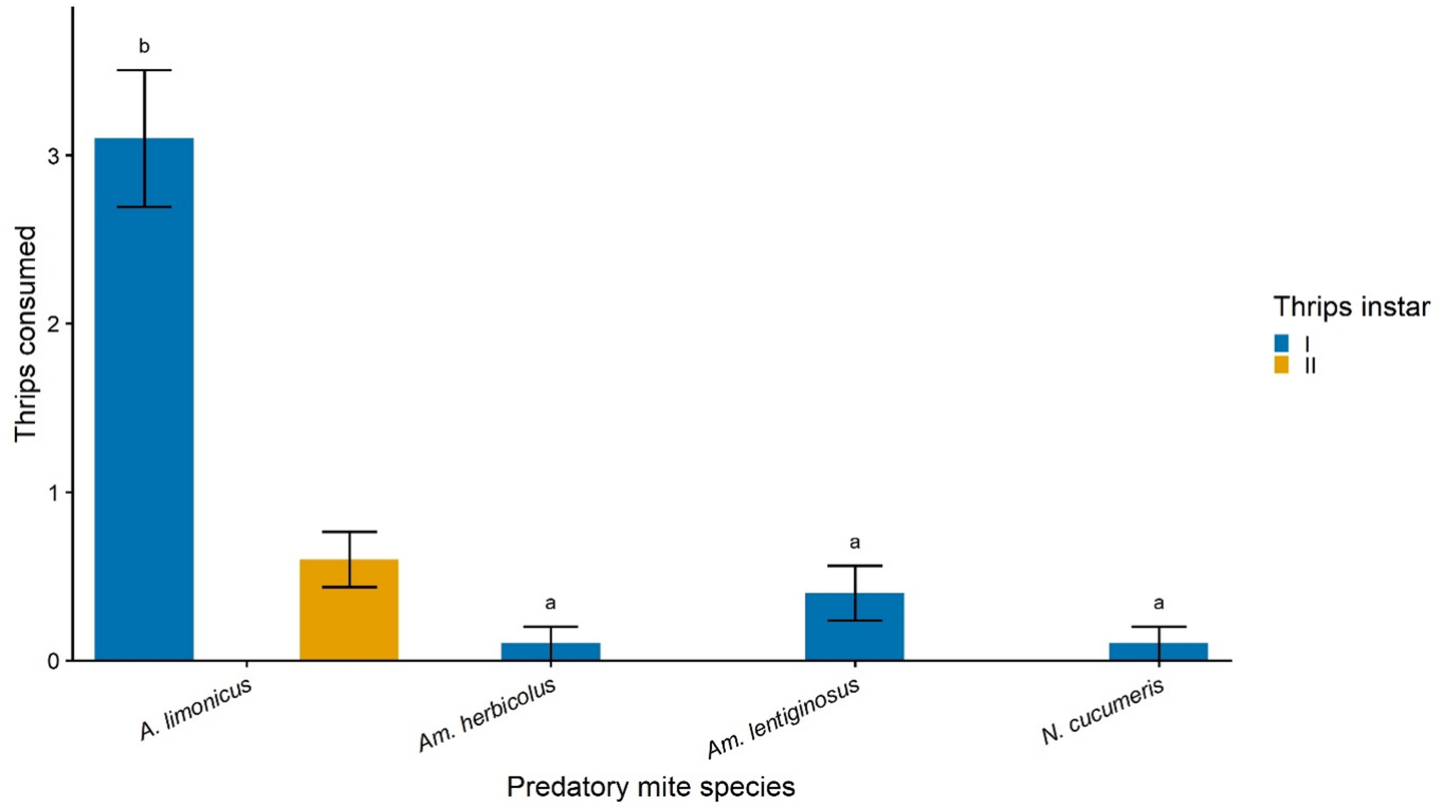
No-choice test. Mean (± SEM) number of first-instar (I) and second-instar (II) larvae of *Heliothrips haemorrhoidalis* consumed by a female of four predatory mite species (*Amblydromalus limonicus*, *Amblyseius herbicolus*, *Amblyseius lentiginosus*, and *Neoseiulus cucumeris*) over a 24 h period. Each treatment consisted of five thrips larvae offered per predator, with 10 replicates per treatment. Different lowercase letters indicate significant differences among predatory mite species within the first-instar treatment (ART ANOVA pairwise comparisons, *p* < 0.05). No letters are shown for the second-instar treatment because only *A. limonicus* consumed second-instar larvae, whereas *Am. herbicolus*, *Am. lentiginosus*, and *N. cucumeris* consumed none in any replicate

In this experiment, the predatory mite species from no-choice test that showed the highest capacity for feeding on both instars of thrips larvae (in our case, *A. limonicus*—see Results, Fig. 1) was simultaneously offered two developmental stages of thrips (I and II) to assess prey stage preference. Each experimental arena contained a mixed prey treatment consisting of equal numbers (*n* = 5) of first- and second-instar thrips.

A single young predatory mite (< 7 d after emergence; mated; starved for one day) was introduced into each arena at the beginning of the experiment. Ten independent replicates were conducted for this choice test (Xin and Zhang 2021). At the end of the 24 h exposure period, the number of thrips consumed from each instar was recorded for each replicate. Prey consumption data were subsequently used to quantify stage-specific predation and to calculate preference indices.

### Functional response analysis

For each experimental cell, a single predator individual (*A. limonicus*) was exposed to a fixed number of prey, and the number of prey consumed within 24 h was recorded. Initial prey numbers (*N₀*) were at six levels *(n* = 1, 2, 4, 6, 8, and 10 individuals), with 10 replicates prey density, giving a total of 60 observations. Each prey density treatment was analysed as an independent set of observations. To determine the type of functional response, we first analysed the proportion of prey consumed using logistic regression. Then, a binomial generalised linear model with a logit link function was fitted to the data, using the number of prey consumed and remaining prey as the response variables and initial prey density as the explanatory variable. Both linear and quadratic terms of prey density were included in the model. A significantly negative linear coefficient combined with a non-significant quadratic term was interpreted as evidence for a Type II functional response, whereas a significantly positive linear term followed by a significantly negative quadratic term would indicate a Type III functional response.

### Statistical analysis

We used the software R, version 4.4.0 (R Core team 2024) for all statistical analyses in this study. Results are summarised as means and standard errors of the mean (SEM). Because the prey consumption data did not meet the assumptions of normality, we applied an aligned rank transform (ART) analysis of variance (ANOVA) using the ARTool package to test the effects of predator species, prey stage, and their interaction in a factorial framework (Kay and Wobbrock 2021).

We used ART ANOVAs to compare the number of thrips consumed among different predatory mite species, thrips developmental stages, and their interaction. When significant effects were detected, post hoc comparisons were conducted using estimated marginal means derived from the ART model with appropriate adjustment for multiple comparisons (Kay and Wobbrock 2021).

A significance level of *p* < 0.05 was applied for all statistical tests. To quantify prey stage preference in the choice test, where first- and second-instar thrips were offered simultaneously, Manly’s preference index (Manly’s α) was calculated for each replicate. Manly’s α values range from 0 to 1, with a value of 0.5 indicating no preference between prey stages, values greater than 0.5 indicating preference for first-instar thrips, and values less than 0.5 indicating preference for second-instar thrips (Manly et al. 2002). Preference indices were summarised using means ± SEM to describe overall trends in prey selection.

In addition to the logistic regression approach, a generalised non-replacement functional response model incorporating a flexible scaling exponent (*q*) was fitted using the *frair_fit()* function in the R package *frair* (Pritchard et al. 2017) to further evaluate potential deviations from a Type II response. In this framework, a Type II functional response is represented by the special case where *q* = 0, whereas *q* > 0 indicates a tendency towards a Type III-like response. Models allowing *q* to be freely estimated were compared with nested models in which *q* was fixed at 0 using Akaike’s information criterion (AIC). Differences in AIC (ΔAIC) were calculated as AIC(*q* free) − AIC(*q* = 0) and used to assess whether inclusion of the scaling exponent improved model fit. Positive values of ΔAIC indicate that allowing *q* to vary did not sufficiently improve model fit to justify the more complex model, whereas negative values support deviation from a Type II response.Attack rate (*a*) and handling time (*h*) were estimated by fitting Rogers’ random predator equation for non-replacement experiments using *frair_fit()* with response = “*rogersII*” (Pritchard et al. 2017):$\:{N}_{e}={N}_{0}\left[1-\mathrm{exp}\left(a\left(h{N}_{e}-T\right)\right)\right]$

where *Nₑ* is the number of prey consumed, *N₀* is the initial prey level, *a* is the attack rate, *h* is the handling time, and *T* is the total experimental duration (1 day = 24 h). Handling time was expressed as a proportion of the 24 h experimental period. All functional response analyses were conducted in R, and parameter estimates were derived separately for each predator-prey dataset.

## Results

In the no-choice test, predation rates differed significantly among predatory mite species and between thrips larval instars (Fig. 1; Table 1). The ART ANOVA revealed significant main effects of predatory mite species (F₃,₇₂ = 28.15, *p* < 0.001) and thrips instars (F₁,₇₂ = 72.08, *p* < 0.001), as well as a significant mite species × thrips stage interaction (F₃,₇₂ = 23.25, *p* < 0.001), indicating that the effect of prey stage on consumption varied among predator species. When first-instar larvae were offered, *A. limonicus* consumed significantly more prey than *N. cucumeris*, *Am. herbicolus*, and *Am. lentiginosus* (all *p* < 0.01), whereas no significant differences were detected among the latter three species (all *p* > 0.99). Mean consumption of first-instar larvae was 3.10 ± 0.41 for *A. limonicus*, compared with 0.10 ± 0.10 for *Am. herbicolus*, 0.40 ± 0.16 for *Am. lentiginosus*, and 0.10 ± 0.10 for *N. cucumeris* (Table 1). When second-instar larvae were offered, *A. limonicus* again consumed significantly more prey than the other three species (all *p* < 0.001), with a mean consumption of 0.60 ± 0.16 larvae. In contrast, *Am. herbicolus*, *Am. lentiginosus*, and *N. cucumeris* consumed no second-instar larvae in any replicate (Table 1). Because only *A. limonicus* consumed both larval stages, subsequent choice and functional response experiments were conducted only for this species.

**Table 1:** No-choice test. Mean (± SEM) consumption of first (I) and second instar (II) *Heliothrips haemorrhoidalis* by four predatory mite species over a 24 h period. Each replicate consisted of five thrips offered per predator, with 10 replicates per treatment. Lowercase letters (a, b) following the means followed by different letters indicate significant differences (ART ANOVA pairwise comparisons: *p* < 0.05). A slash (/) indicates treatments in which no prey were consumed. A—*Amblydromalus; Am—Amblyseius. N—Neoseiulus*

Predatory mite	Thrips stage	*n*	Thrips consumed
*A. limonicus*	I	10	3.10 ± 0.41^b^
	II	10	0.60 ± 0.16
*Am. herbicolus*	I	10	0.10 ± 0.10^a^
	II	10	/
*Am. lentiginosus*	I	10	0.40 ± 0.16^a^
	II	10	/
*N. cucumeris*	I	10	0.10 ± 0.10^a^
	II	10	/

In the choice test, *A. limonicus* consumed only first-instar thrips, with a mean consumption of 1.9 ± 0.38 individuals over 24 in all ten replicates. Our Manly’s preference index indicated a complete preference for first-instar prey (α_I = 1.00; α_II = 0.00).

In the final functional response experiment, a binomial logistic regression based on proportional prey consumption showed a significant negative linear effect of prey density on the proportion of prey consumed (*b* = − 0.617 ± 0.301, *z* = − 2.05, *p* = 0.04), indicating that the proportion of prey consumed declined as initial prey density increased. No significant quadratic term was detected, providing no evidence for a Type III functional response.

Consistent with this regression result, the generalised functional response model estimated a scaling exponent (*q*) close to zero (*q* ≈ 0). Allowing *q* to vary did not improve model fit relative to the nested Type II model with *q* fixed at zero (ΔAIC ≤ 2). Together, these results provide no support for a deviation from a Type II functional response, indicating that the proportion of prey consumed declined monotonically with increasing prey density.

**Fig. 2:**
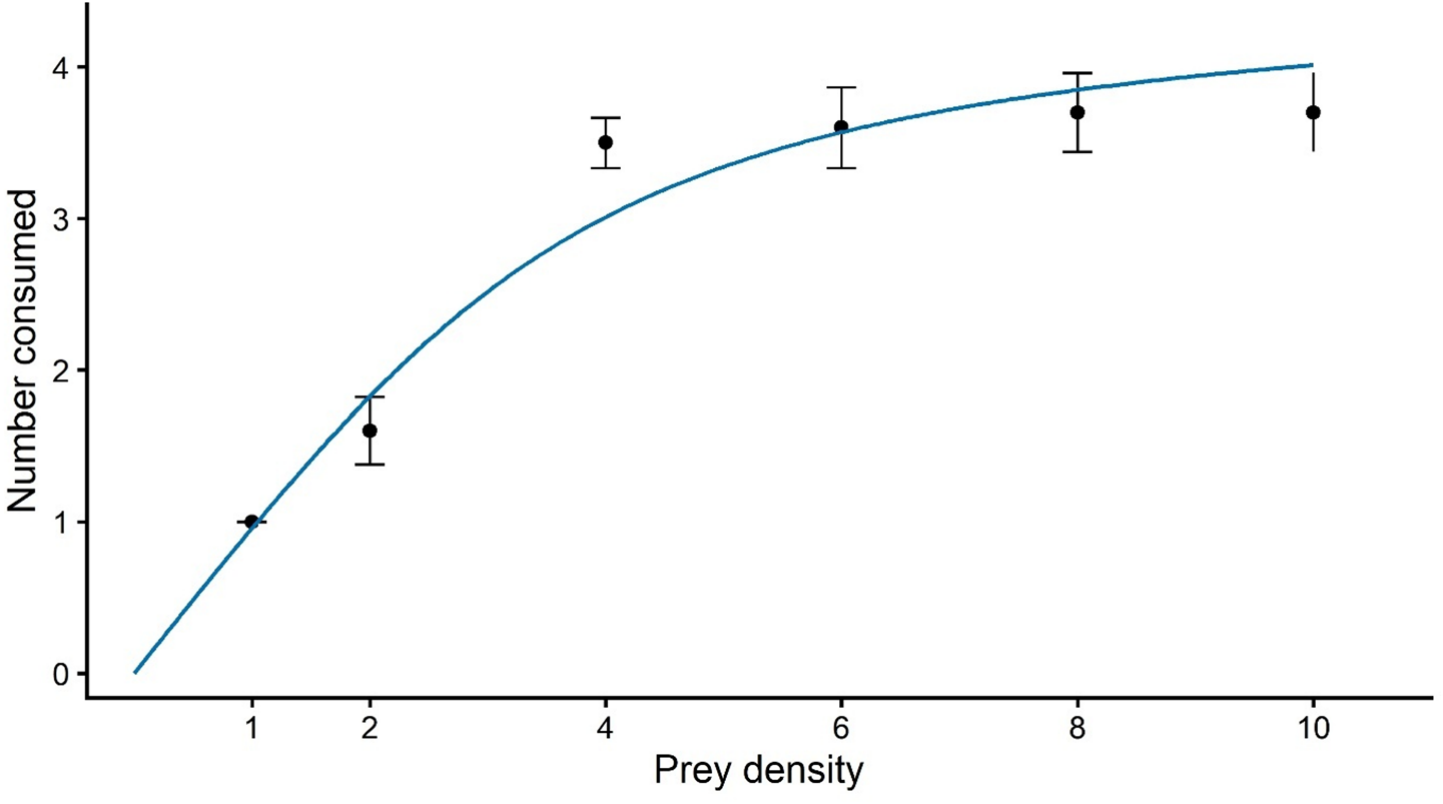
Functional response of the predator (*Amblydromalus limonicus*) to increasing prey (first instar larva *Heliothrips haemorrhoidalis*) density. Our observed prey consumption and fitted Type II functional response curve is based on the Rogers random predator equation showing the relationship between initial prey density (*N₀*) and the number of prey consumed (*Nₑ*) over a 24 h period

Parameter estimation using Rogers’ Type II functional response model yielded a positive attack rate (*a* = 4.05 day⁻¹) and a handling time of *h* = 0.218 days (approximately 5.23 h). Predation rate increased rapidly at low prey densities but approached an asymptote at higher densities, consistent with handling time constraints (Fig. 2).

## Discussion

Among the four predatory mites tested, *A. limonicus* exhibited the highest predation rates overall and showed a pronounced preference for first-instar larvae of *H. haemorrhoidalis*. This pattern is consistent with results from other thrips systems. For example, on greenhouse cucumber infested with western flower thrips, *Frankliniella occidentalis*, *A. limonicus* performed better than *N. cucumeris* and was identified as one of the most effective phytoseiid predators among the species tested (Messelink et al. 2006). Similarly, on immature *Sericothrips staphylinus*, *A. limonicus* showed the highest predation and oviposition rates, *Am. herbicolus* showed intermediate performance, and *N. cucumeris* the lowest (Lam et al. 2019). These studies, together with earlier work showing effective predation by phytoseiids on early thrips stages (Cloutier and Johnson 1993; Shipp and Whitfield 1991), support the view that early larval stages of thrips are generally more vulnerable to predation by phytoseiids than later stages. The ability of *A. limonicus* to consume both larval stages, albeit at different rates, suggests that it may contribute to suppression of greenhouse thrips populations during early population build-up. However, because the present study was based on short-term laboratory assays, these results should be interpreted cautiously and do not by themselves demonstrate effective biological control under orchard conditions.

In contrast, *Am. lentiginosus* displayed moderate predation on first-instar thrips but negligible consumption of second instars. Although its mean consumption of first instars was higher than that of second instars, this difference was not statistically significant. Due to the very low rates of prey predation, no further experiments on functional response were conducted on this species.

Both *N. cucumeris* and *Am. herbicolus* exhibited very low predation rates on thrips regardless of prey stage, feeding very little on first-instar larvae and not feeding on second-instar larvae under the present conditions. These results suggest that, under the conditions tested, these species are unlikely to contribute substantially to direct thrips suppression. Low consumption rates may indicate that greenhouse thrips larvae, particularly second-instar larvae, were poor prey for these two predatory mite species under the experimental conditions used here.

The selective predation (i.e. choice) experiment demonstrated a pronounced preference for first instar larvae by *A. limonicus* when first- and second-instar thrips were offered simultaneously. This observed preference was probably driven by stage-specific differences in prey vulnerability and defence. First-instar thrips are smaller, less mobile, and possess less developed defensive capabilities compared with later instars, which may reduce handling time and increase capture success for *A. limonicus*. In contrast, second-instar thrips are larger and more mobile, potentially increasing escape rates and handling costs. When both prey stages were simultaneously available, these differences may have contributed to the absence of predation on second-instar larvae in the choice test (Reitz et al. 2006; Rosenheim et al. 1993).

From an applied perspective, the bias of *A. limonicus* towards early thrips instars suggests that its effectiveness as a biological control agent is likely to be greatest when predator populations are established early, coinciding with the presence of young thrips cohorts. Such stage-specific predation may contribute to suppression of thrips population growth by targeting the most vulnerable life stages before they reach reproductive maturity.

The first- and second-instar larvae of *H. haemorrhoidalis* are known to secrete a viscous substance that accumulates on leaf surfaces as thrips density increases. Such secretions may contaminate the foraging substrate and hinder the movement of small arthropod predators, thereby reducing their ability to effectively locate, capture, or subdue prey, and may also function as a defensive mechanism. Previous studies have demonstrated that this viscous material can entangle and even kill predatory mites such as *N. californicus* under laboratory conditions (Sazo et al. 2006). The accumulation of these secretions (per unit leaf area) is likely to be greater at high prey densities, which might have contributed to reduction in the proportion of prey consumed observed at the highest prey levels (i.e. densities) in the present study (Cao et al. 2026). However, this pattern was also likely influenced by predator satiation and handling-time limitation, because under a Type II functional response the number of prey consumed is expected to approach an asymptote as prey density increases.

### Limitations and caveats

This study was conducted under relatively idealised laboratory conditions, using a small experimental set-up and observing predation over only 24 h, which differs from the actual environment of avocado plants. In real plants, leaf structures are more complex, with microclimate changes and various hiding places, all of which affect predators’ chances of finding prey and the probability of successful predation. The experiment covered only a 24 h period and did not assess multi-day population dynamics or oviposition. Furthermore, the use of a single-predator design did not allow evaluation of intraspecific interference or other density-dependent interactions among predators.

The functional response experiment set up had only a single predator and did not consider mutual interference among predators common in greenhouses or orchards, intraspecific competition, or population changes over longer time scales. Future research should be conducted under conditions closer to actual production environments, such as greenhouse experiments on whole avocado plants, extending the observation period, and evaluating their sustained control capabilities and long-term suppression effects on thrips populations through multiple predator releases. It would also be desirable and possible to try combine releases with larger insect predators (e.g. lacewing larvae; see Cao et al. 2026) that prefer to prey on later-stage larvae or adults to test if complementary predation at different stages can lead to better overall control. Future studies could also investigate egg-stage interactions, including whether predatory mites can locate and prey on *H. haemorrhoidalis* eggs embedded in leaf tissue, as well as their multi-day predation and oviposition performance in arenas containing leaf material.

## Conclusion

This study has demonstrated marked interspecific variation in predation capacity among phytoseiid mites feeding on the greenhouse thrips, with predation capacity strongly affected by prey developmental stage. Among the four species tested, *A. limonicus* consistently exhibited the highest predation rates and a clear preference for first-instar thrips across no-choice, choice, and functional response experiments, whereas *Am. herbicolus*, *Am. lentiginosus*, and *N. cucumeris* showed minimal predation, suggesting a limited role in direct thrips suppression under the conditions tested. The exclusive targeting of early instars by *A. limonicus*, together with its Type II functional response, indicates that prey vulnerability and handling constraints are key drivers of predator-prey interactions, with greatest efficacy at low to moderate thrips densities. Collectively, these results highlight that effective biological control of thrips in avocado orchards depends on predator species identity and prey stage and indicate that *A. limonicus* is worthy of further evaluation as a potential biological control agent under more realistic conditions, including experiments with leaf tissue, longer observation periods, and greenhouse or semi-field settings. At present, evidence for its practical use in orchard IPM remains limited.

## Data Availability

The dataset is for open access from: https://datastore.landcareresearch.co.nz/dataset/raw-data-mite-thrips.
